# Interaction with TopBP1 Is Required for Human Papillomavirus 16 E2 Plasmid Segregation/Retention Function during Mitosis

**DOI:** 10.1128/jvi.00830-22

**Published:** 2022-07-26

**Authors:** Apurva T. Prabhakar, Claire D. James, Dipon Das, Christian T. Fontan, Raymonde Otoa, Xu Wang, Molly L. Bristol, Iain M. Morgan

**Affiliations:** a Virginia Commonwealth University (VCU), Philips Institute for Oral Health Research, School of Dentistry, Richmond, Virginia, USA; b VCU Massey Cancer Center, Richmond, Virginia, USA; University of Toronto

**Keywords:** E2, TopBP1, chromosome segregation, papillomavirus, plasmid retention

## Abstract

Human papillomavirus 16 (HPV16) E2 is a DNA-binding protein that regulates transcription, replication and potentially, segregation of the HPV16 genome during the viral life cycle. In the segregation model, E2 simultaneously binds to viral and host chromatin, acting as a bridge to ensure that viral genomes reside in daughter nuclei following cell division. The host chromatin receptor for E2 mediating this function is unknown. Recently, we demonstrated that CK2 phosphorylation of E2 on serine 23 (S23) is required for interaction with TopBP1, and that this interaction promotes E2 and TopBP1 recruitment to mitotic chromatin. Here, we demonstrate that in U2OS cells expressing wild-type E2 and a non-TopBP1-binding mutant (S23A, serine 23 mutated to alanine), interaction with TopBP1 is essential for E2 recruitment of plasmids to mitotic chromatin. Using novel quantitative segregation assays, we demonstrate that interaction with TopBP1 is required for E2 plasmid segregation function in U2OS and N/Tert-1 cells. Small interfering RNA (siRNA) knockdown of TopBP1 or CK2 enzyme components disrupts E2 segregation/retention function. The interaction of E2 with TopBP1 promotes increased levels of E2 protein during mitosis in U2OS and N/Tert-1 cells, as well as in human foreskin keratinocytes (HFK) immortalized by the HPV16 genome. Overall, our results demonstrate that E2 has plasmid segregation activity, and that the E2-TopBP1 interaction is essential for this E2 function.

**IMPORTANCE** HPV16 causes 3% to 4% of all human cancers. It is proposed that during the viral life cycle, the viral genome is actively segregated into daughter nuclei, ensuring viral replication in the subsequent S phase. The E2 protein potentially bridges the viral and host genomes during mitosis to mediate segregation of the circular viral plasmid. Here, we demonstrate that E2 has the ability to mediate plasmid segregation, and that this function is dependent upon interaction with the host protein TopBP1. Additionally, we demonstrate that the E2-TopBP1 interaction promotes enhanced E2 expression during mitosis, which likely promotes the plasmid segregation function of E2. Overall, our results present a mechanism of how HPV16 can segregate its viral genome during an active infection, a critical aspect of the viral life cycle.

## INTRODUCTION

Human papillomaviruses are causative agents in around 5% of all human cancers, targeting anogenital and oropharyngeal regions, with human papillomavirus 16 (HPV16) being the most prevalent type detected (1). There are no drugs for the treatment of HPV cancers that directly target a viral process, and an enhanced understanding of the viral life cycle is required in order to identify novel therapeutic targets. HPVs infect basal epithelial cells, and following cell division, the viral genomes enter the host nuclei, a cellular compartment required for replication of the double-stranded DNA viral genome (2–4). Following entry into the infected cell nucleus, host factors activate transcription from the viral genome (5). The E6 and E7 viral proteins target and degrade p53 and pRb (among other targets), respectively, promoting cellular proliferation and entry into S phase (6).

During S phase, two viral proteins (E1 and E2) interact with host factors to replicate the viral genome (7, 8). The carboxyl-terminal domain of E2 forms homo-dimers and binds to 12-bp palindromic target sequences on the viral long control region (LCR), which controls transcription and replication of the viral genome. Three of the E2 target sequences surround the viral origin of replication, and following binding, E2 recruits the E1 protein to the origin of replication via a protein-protein interaction (9, 10). E1 then interacts with a number of cellular replication factors in order to replicate the viral genome (11–16). In addition to promoting viral replication, E2 has other functions during the viral life cycle. E2 can regulate transcription from the viral genome, activating or repressing depending on the levels of E2 protein (17). E2 can also regulate transcription from the host genome, which has direct relevance to the viral life cycle (18, 19). The final proposed function for E2 during the viral life cycle is acting as a viral segregation factor (20). During mitosis, there is an active mechanism to retain the viral genome in the resulting daughter nuclei. It is proposed that E2 acts as a bridge between host and viral genome to ensure localization of the viral genomes in daughter nuclei following cell division. For bovine papillomavirus type 1 (BPV1) E2, BRD4 is the mitotic chromatin receptor (21–24). However, for high-risk HPV types (those that cause cancer, including HPV16), there have been reports that E2 and BRD4 do not colocalize on mitotic chromatin, suggesting that BRD4 is not the primary mitotic chromatin receptor for these HPV E2 proteins (25, 26). However, there are other reports demonstrating that HPV16 E2 and BRD4 do colocalize on mitotic chromatin (27, 28). Therefore, the role of BRD4 in mediating HPV16 E2 association with mitotic chromatin remains unresolved.

We previously identified an interaction between HPV16 E2 (here, E2) and TopBP1 (29, 30). This interaction is involved in regulating E1-E2 replication function (31–33). TopBP1 is also a strong candidate for mediating the plasmid segregation function of E2. TopBP1 is a multifunctional protein encoding 9 BRCA1 C-terminal (BRCT) domains, and is involved in all aspects of nucleic acid metabolism (34). TopBP1 is also highly functional during mitosis as it can prevent transmission of damaged and under-replicated DNA to daughter cells (35–42). Furthermore, TopBP1 regulates the ability of E2 to interact with interphase chromatin, and colocalizes with E2 on mitotic chromatin (43). Recently, we demonstrated that phosphorylation of E2 on serine 23 promotes a direct interaction between these two proteins *in vitro* and *in vivo*, and that E2 recruits TopBP1 onto mitotic chromatin (44). Our work showed that mutation of serine 23 to alanine (S23A) resulted in a compromised interaction of E2 with mitotic chromatin, and that E2-TopBP1 interaction is essential for the HPV16 life cycle (44).

Building on this, we report here that E2 recruits plasmid DNA to mitotic chromatin in a TopBP1 interaction-dependent manner in U2OS cells. We describe novel quantitative assays demonstrating that the E2-TopBP1 interaction is required for E2 segregation/retention function in both U2OS cells and the human foreskin keratinocyte cell line N/Tert-1. We also demonstrate that in U2OS, N/Tert-1 and human foreskin keratinocytes (HFK) immortalized by HPV16 (HFK+HPV16), the interaction between E2 and TopBP1 is required for increasing both E2 and TopBP1 protein levels during mitosis. While additional host proteins may contribute to the plasmid segregation function of E2, we have demonstrated that the E2-TopBP1 interaction is critical for this function.

## RESULTS

### Development of functional assays for investigating E2 segregation function.

**FIG 1:**
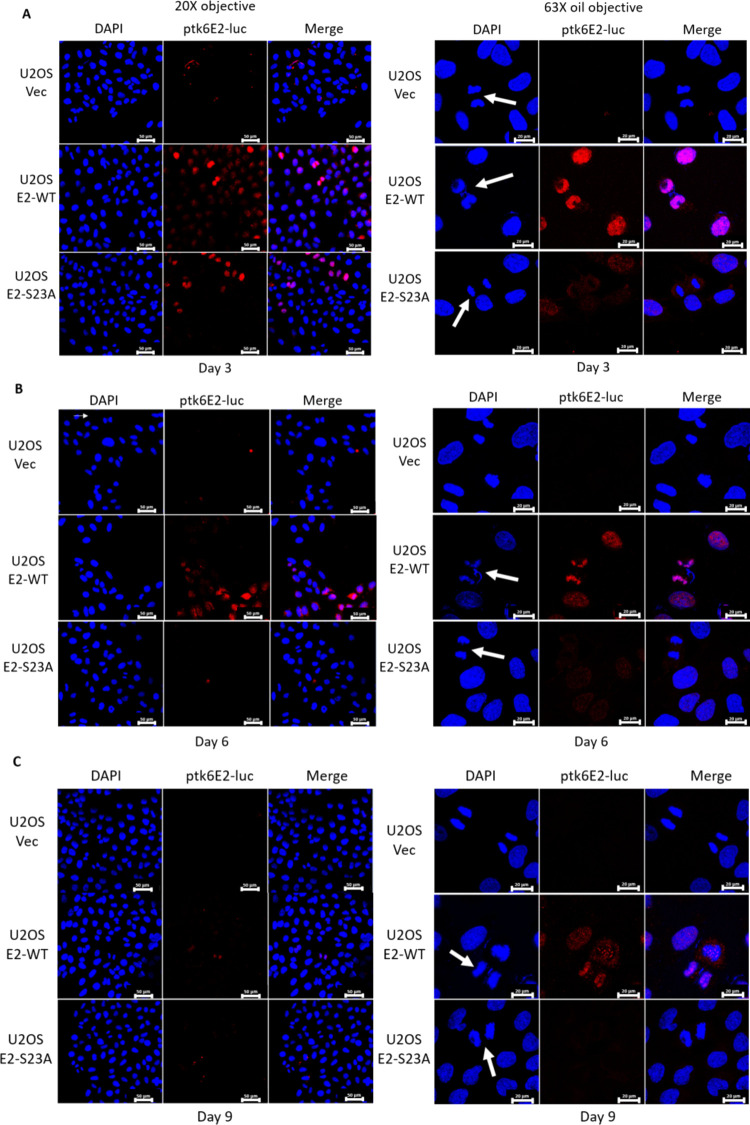
E2 recruits E2 binding site plasmids to U2OS cell mitotic chromatin in a TopBP1 interacting dependent manner. (A) Fluorescently labeled ptk6E2-luc was transfected into the indicated cell lines. Three days later, images were captured by either the Keyence imaging system (BZ-X810) (left) or using a Zeiss LSM700 confocal (right). Mitotic cells are indicated with white arrowheads in the confocal images. (B) Cells shown in panel A were trypsinized and re-plated, and images captured 6 days following transfection, as in panel A (C) Cells shown in panel B were trypsinized and re-plated, and images captured 9 days following transfection, as in panel A.

**FIG 2:**
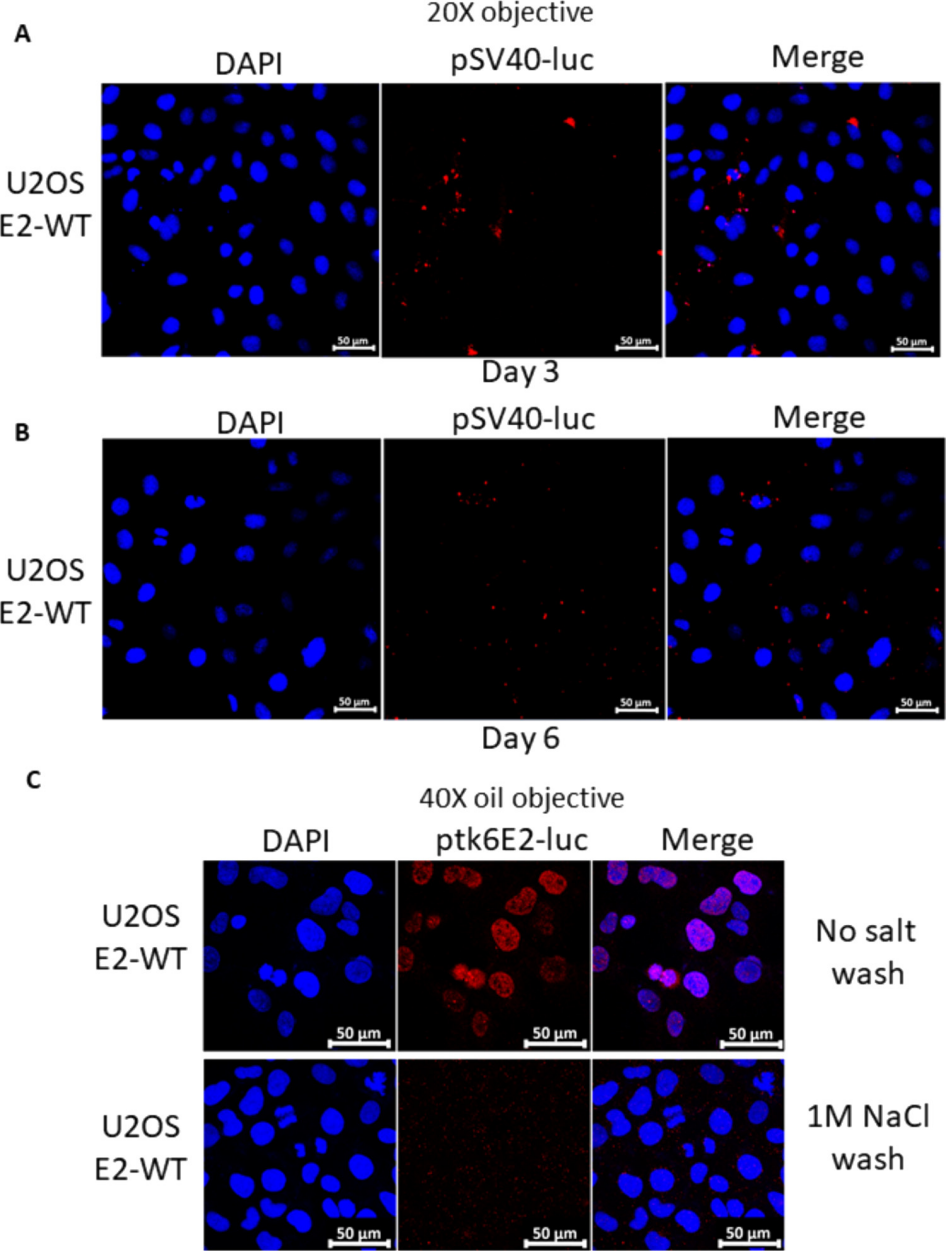
Recruitment of fluorescent plasmids to mitotic chromatin is E2 DNA-binding site-dependent and is not due to integration of the plasmid. (A and B) U2OS E2-WT (WT, wild-type) cells were transfected with fluorescently labeled pSV40-luc (pGL3-Control from Promega, containing the SV40 promoter and enhancer regions but no E2 DNA-binding sites), and 3 days (A) or 6 days (B) following transfection, the Keyence imaging system was used to capture images. (C) U2OS E2-WT cells were transfected with fluorescently labeled ptk6E2-luc and, 3 days later, cells were imaged using a Keyence imaging system following mock treatment (top panels) or a 1 M NaCl wash (bottom panels).

Previously, we demonstrated colocalization of E2 and TopBP1 on mitotic chromatin and showed that E2 can recruit TopBP1 to mitotic chromatin (43, 44). There were no assays available to determine whether E2 recruits plasmids onto mitotic chromatin; therefore, we developed an assay. Using Label IT Tracker (Mirus, cat no. MIR7025), we covalently added a fluorescent tag to ptk6E2-luc, a plasmid containing 6 E2 DNA-binding sites upstream from the luciferase gene controlled by the tk promoter (45). We transfected this labeled plasmid into U2OS-Vec (vector control), U2OS-E2-WT (stably expressing wild-type E2), and U2OS-E2-S23A (stably expressing E2 with serine 23 mutated to alanine, disrupting interaction with TopBP1) cell lines. Three days later, we fixed the transfected cells and looked for the presence of the fluorescent ptk6E2-luc (Fig. 1A, left panel). There was a notable nuclear presence of the labeled plasmid in U2OS-E2-WT and U2OS-E2-S23A, but not in the U2OS-Vec cells. This suggests that both E2WT and E2S23A can bind to the labeled plasmid and retain it in the nucleus (as E2 is a nuclear protein). Both E2WT and E2S23A activate transcription to identical levels in a 3-day transcription assay in U2OS cells (44). However, it was noticeable that in U2OS-E2-WT mitotic cells, but not in U2OS-E2-S23A mitotic cells, the fluorescent ptk6E2-luc was retained on the mitotic chromatin (mitotic cells indicated with white arrows). To investigate this further, confocal images were taken at a higher resolution (Fig. 1A, right panel). As expected, no fluorescence was detected on mitotic U2OS-Vec cells (top panels). However, in U2OS-E2-WT cells, there was a clear retention of the fluorescent plasmid on the mitotic chromatin (middle panels), while in U2OS-E2-S23A cells this phenotype was absent (bottom panels). Retention of fluorescent ptk6E2-luc was detectable on mitotic chromatin in repeated U2OS-E2-WT cells, but never in U2OS-E2-S23A cells. To determine whether this plasmid recruitment to mitotic chromatin by E2-WT promoted retention of the plasmid in transfected cells we trypsinized these cells, re-plated them, and investigated fluorescence at day 6 following transfection (Fig. 1B). The left panel demonstrates significant retention of the plasmid in E2-WT cells compared with that in Vec-control (no staining) and E2-S23A. Confocal images demonstrate that at day 6, the fluorescent DNA is still interacting with mitotic chromatin. This process was repeated and fluorescence investigated at day 9 following transfection (Fig. 1C). There remained detectable fluorescent plasmid in the E2-WT cells (left and right panels) and no detectable fluorescence in Vec-control of E2-S23A cells. Presence of the fluorescent plasmid on mitotic chromatin was still detectable in the E2-WT cells (right panel). To confirm that recruitment of the plasmid to mitotic chromatin by E2-WT was not due to random interaction with the fluorescently labeled DNA, we demonstrated that pSV40-luc (pGL3-CONT from Promega, which has SV40 promoter and enhancer regions driving luciferase expression) was not retained in the nuclei or located to mitotic chromatin in U2OS-E2-WT cells (Fig. 2A), and that most detectable fluorescent DNA was lost by day 6 (Fig. 2B). To confirm that E2-WT was not promoting integration of the fluorescent plasmid into the host genome, we washed the fixed cells with 1 M salt. Because this wash breaks noncovalent interactions and removes the fluorescent DNA from the chromatin of U2OS-E2-WT cells, we concluded that the plasmid had not become integrated (Fig. 2C). In these experiments, we could not co-stain for E2 protein because the permeabilization disrupted the interaction of the fluorescent plasmids with the chromatin. These cell lines stably express E2, and most cells retain E2 protein expression (44).

**FIG 3:**
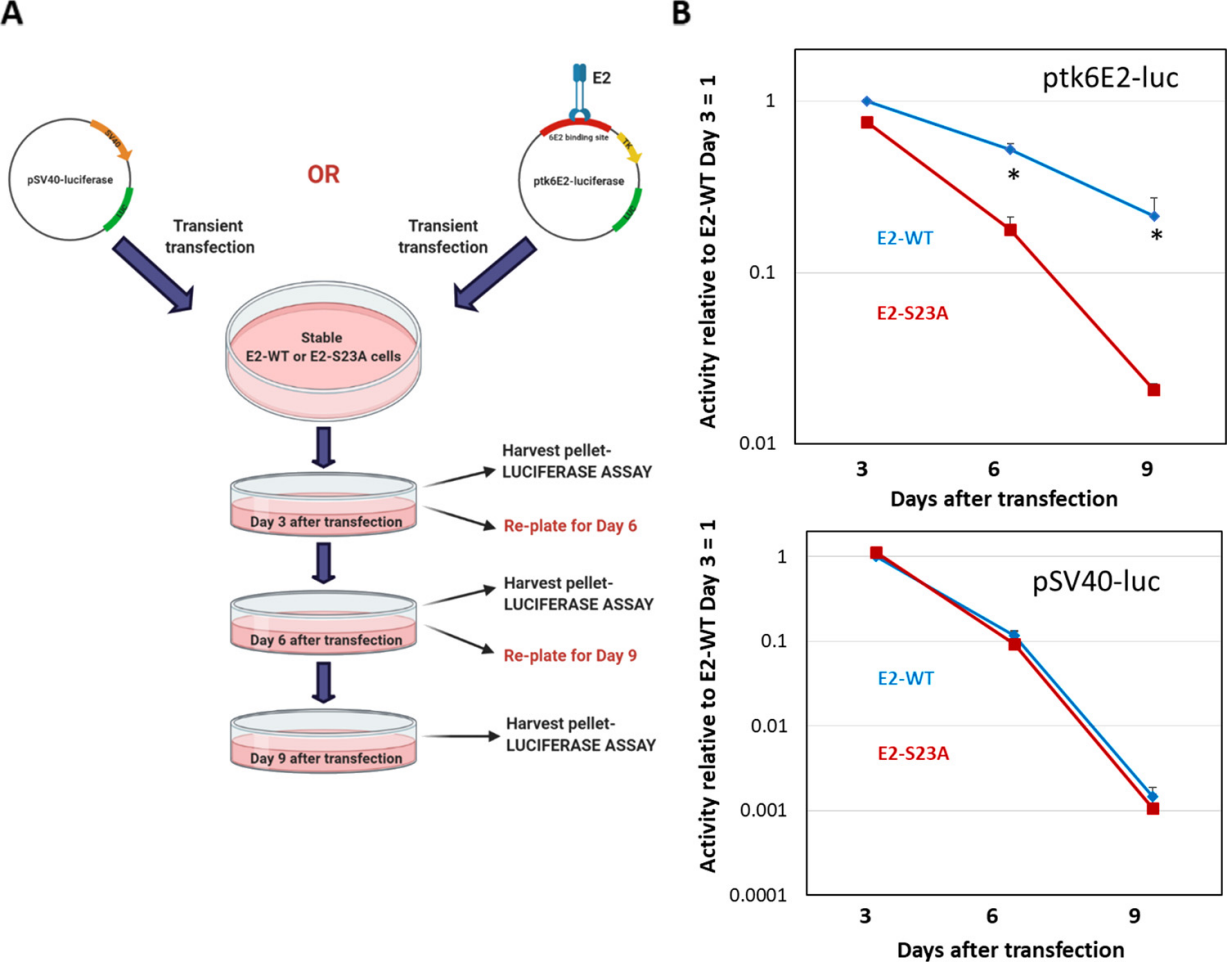
Plasmid retention/segregation by E2 in U2OS cells. (A) Our luciferase-based segregation/retention assay, as summarized in the text. (B) Luciferase activity detected in U2OS cells. Day 3 luciferase activity for both E2-WT and E2-S23A was statistically identical (44). For ptk6E2-luc, at day 6, E2-WT retained around 85% of day 3 activity, while E2-S23A retained around 15%. At day 9, E2-WT retained around 15% of day 3 activity, while most activity was lost with E2-S23A. For pSV40-luc, activity was lost rapidly in both E2-WT and E2-S23A cells. Asterisk (*) indicates a significant difference between this sample and the others on days 6 and 9, *P* < 0.05. Standard error bars are too small to appear on the log scale; results represent the summary of three independent experiments.

When there is no selective pressure present, transfected plasmids are quickly lost from cells after 3 to 4 days, explaining the loss of transfected plasmids over time as shown in in Fig. 1 (46). It occurred to us that this retention of ptk6E2-luc by E2-WT would allow us to quantitate retention by measuring luciferase activity. On this principle, we developed a novel quantitative luciferase-based assay to measure the plasmid segregation/retention function of E2-WT (Fig. 3A). In this assay, ptk6E2-luc or pSV40-luc are transfected independently into either U2OS-E2-WT or U2OS-E2-S23A. We could not use U2OS-Vec cells in this assay because the luciferase activity of ptk6E2-luc is very low in the absence of E2, which made the measurement of plasmid retention via luciferase detection impractical. Three days following transfection, the cells were trypsinized; half were used for a luciferase assay, and the other half were re-plated for culture. At day 6, cells were trypsinized; half of them were used for a luciferase assay, and the other half were re-plated. At day 9, all cells were harvested for luciferase assays. Table 1 details the average luciferase activity detected in triplicate samples from one of these experiments. With ptk6E2-luc, there was no significant difference between the activities in E2-WT and E2-S23A cells on day 3 (Table 2 standardizes the results to E2-WT = 1). However, on days 6 and 9, there was significantly more ptk6E2-luc activity in E2-WT than in E2-S23A cells (Table 2). With pSV40-luc, there was no statistically significant difference in luciferase activity between E2-WT and E2-S23A at any time point (Table 2). We represented these results graphically (Fig. 3B). These results reflect the retention of ptk6E2-luc by E2-WT, as indicated by the fluorescence assays described in Fig. 1.

**TABLE 1:** Average luciferase activity on the indicated days from three independent experiments (used to generate Fig. 3B)^*a*^

Day	ptk6E2-luc		pSV40-luc	
	E2-WT	E2-S23A	E2-WT	E2-S23A
3	283	214.667	8994	10090.8
6	148.3	50.3	1058	827.6
9	60.5	5.8	13	9.5

a E2-WT, stably expressing wild-type E2; E2-S23A, stably expressing E2 with serine 23 mutated to alanine.

**TABLE 2:** Fold change relative to E2-WT at day 3 = 1 from the novel quantitative luciferase-based assay (represented in Fig. 3B)^*a*^

Plasmid	E2-WT (fold change ± SE)	E2-S23A (fold change ± SE)	*P* value
ptk6E2-luc			
Day 3	1 ± 0.018	0.76 ± 0.028	0.0629
Day 6	0.524 ± 0.042	0.178 ± 0.033	0.0003^*b*^
Day 9	0.214 ± 0.060	0.020 ± 0.002	0.0001^*b*^
pSV40-luc			
Day 3	1 ± 0.027	1.122 ± 0.027	0.4427
Day 6	0.117 ± 0.014	0.092 ± 0.040	0.1718
Day 9	0.0014 ± 0.00041	0.001 ± 0.004	0.2932

a E2-WT, stably expressing wild-type E2; E2-S23A, stably expressing E2 with serine 23 mutated to alanine; SE, standard error of the mean.

b Significant difference between E2-WT and E2-S23A.

### TopBP1 interaction is required for the plasmid retention/segregation function of E2.

**FIG 4:**
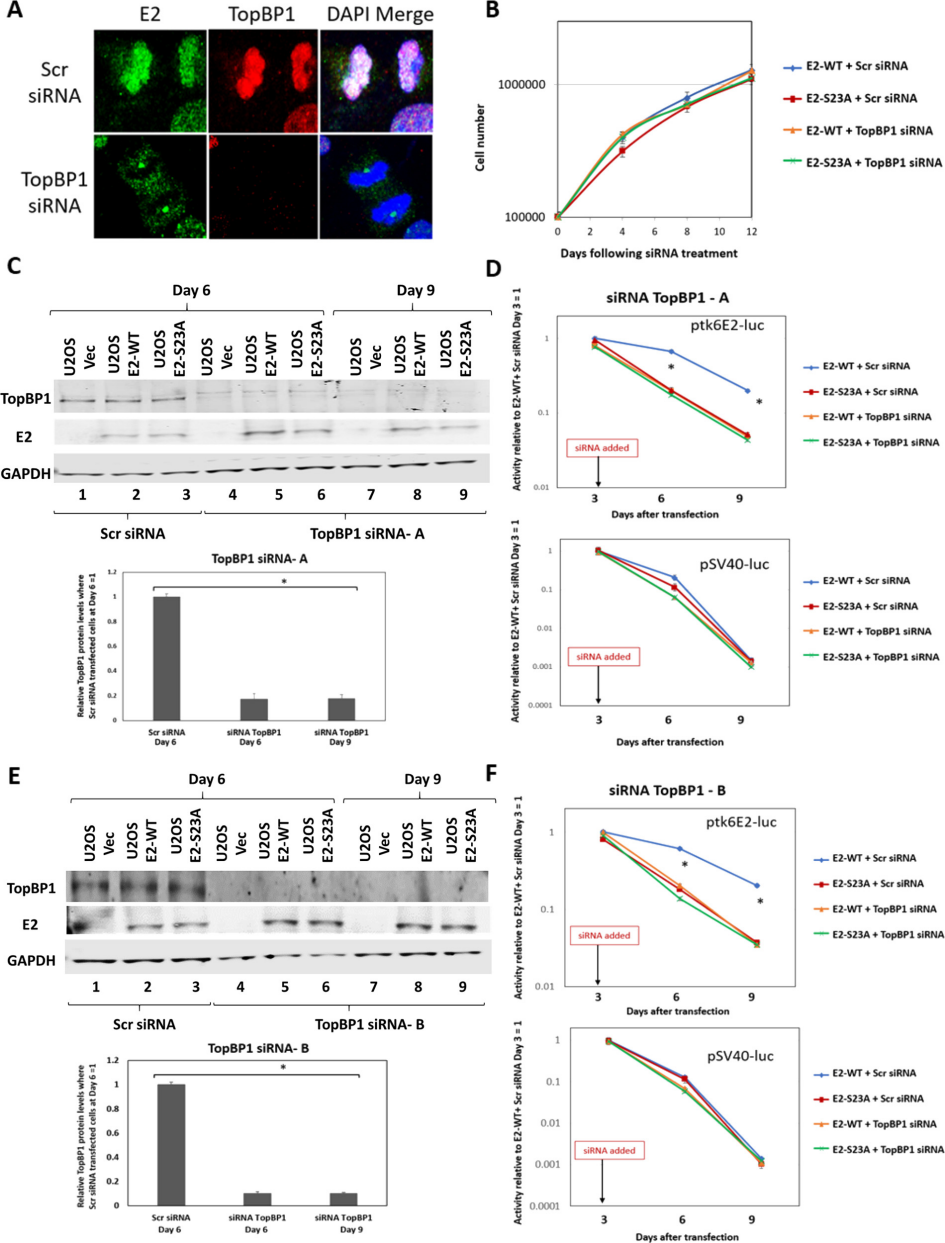
E2 plasmid segregation/retention function is dependent on TopBP1 in U2OS cells. (A) Small interfering RNA (siRNA) knockdown of TopBP1 (bottom panels) removes the interaction of E2-WT with mitotic chromatin. (B) U2OS cells were treated with the indicated siRNAs and their growth measured over the indicated time period. (C) siRNA was added to cells on day 3 of our luciferase based plasmid retention/segregation assay, as described in Fig. 3A. Protein was prepared from cells 6 and 9 days following transfection, and Western blotting was performed for the indicated proteins. TopBP1 siRNA knocked down TopBP1 expression, which persisted from day 6 until day 9. The knockdown was quantitated by averaging out the TopBP1 levels in the three different siRNA treatment sets (bottom panel). (D) Knockdown of TopBP1 expression abolishes the ability of E2-WT to retain ptk6E2-luc. Asterisk (*) indicates a significant difference between this sample and the others on days 6 and 9, *P* < 0.05. Standard error bars are too small to show up on the log scale. (E and F) An alternative TopBP1 siRNA was used to those shown in in panels C and D, and these duplicate experiments demonstrate that the effects of the TopBP1 siRNA were not due to off-target effects.

We set out to confirm that interaction with TopBP1 is essential for the retention/segregation function of E2-WT using our luciferase assay. In order to do this, we knocked out TopBP1 using small interfering RNA (siRNA). Figure 4A confirms that, following TopBP1 knockdown, E2 is removed from mitotic chromatin in U2OS cells; this was observed in multiple mitotic cells. During the time course of the TopBP1 siRNA knockdown, there was no significant alteration of cellular growth due to the absence of TopBP1 (Fig. 4B). We next carried out the assay described in Fig. 3A but introduced TopBP1 or scrambled control siRNA to the cells at the day 3 time point prior to replating. Figure 4C demonstrates that at day 6 following transfection (3 days following addition of siRNA) there was a significant knockdown of TopBP1 (compare lanes 4 to 6, TopBP1 siRNA-A, with lanes 1 to 3, scr siRNA). This knockdown extended to day 9 (lanes 7 to 9). The knockdown was quantitated on days 6 and 9 relative to scrambled siRNA on day 6, demonstrating a significant knockdown of TopBP1 expression. Figure 4D (upper panel) demonstrates that knockdown of TopBP1 expression abrogated the retention of ptk6E2-luc luciferase activity compared with the scr siRNA control. Knockdown of TopBP1 made no difference to the activity detected with E2-S23A at either time point. Similarly, knockdown of TopBP1 had no effect on the activity of pSV40-luc in either of the E2-expressing lines at either time point. We repeated the TopBP1 knockdown with an additional TopBP1 siRNA-B; these experiments produced identical results to those in Fig. 4C and D (Fig. 4E and F).

**FIG 5:**
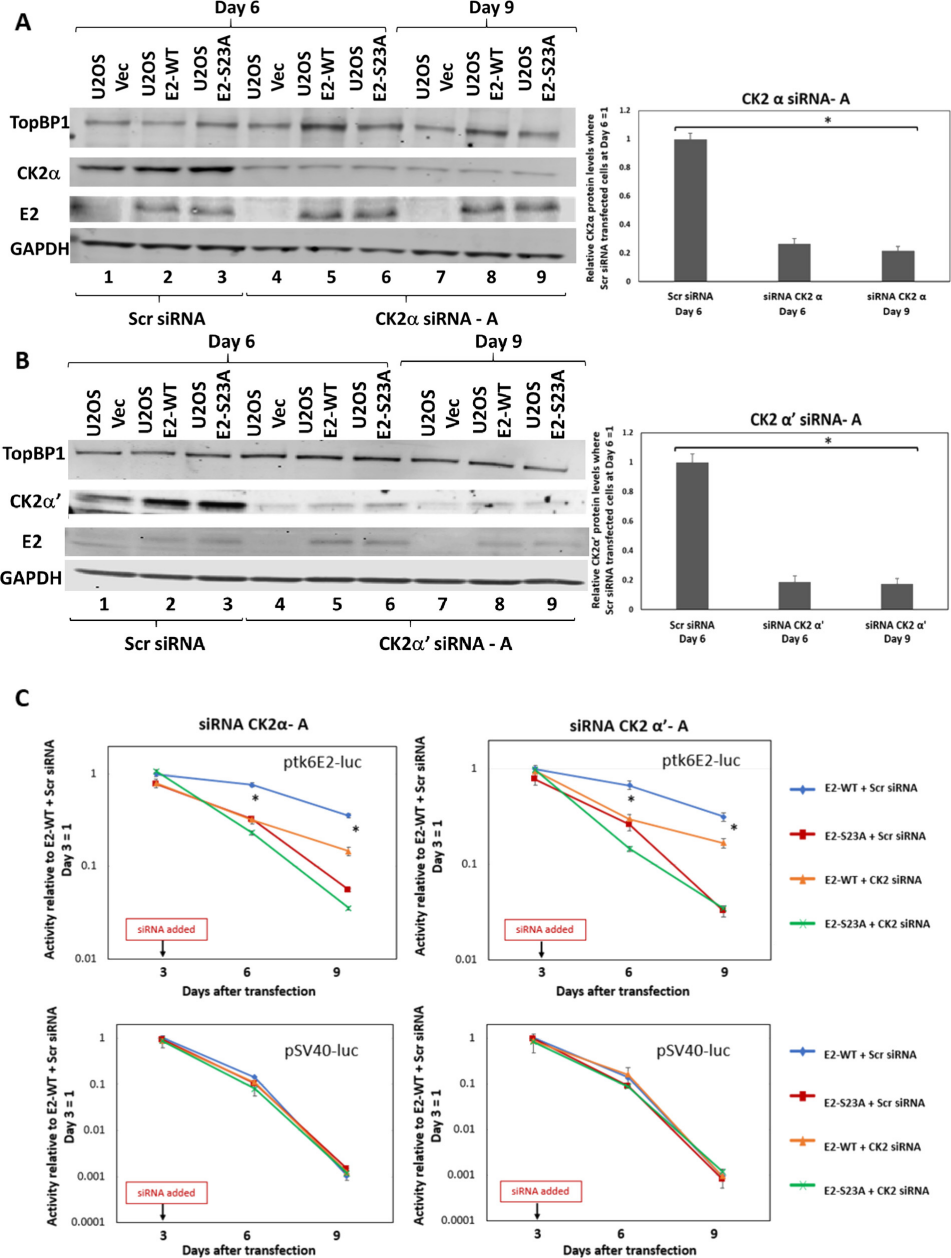
Knockdown of CK2 components disrupts E2 segregation/retention function in U2OS cells. (A and B) Indicated siRNA was added to cells on day 3 of our luciferase-based plasmid retention/segregation assay, as described in Fig. 3A. Protein was then prepared from cells 6 and 9 days following transfection, and Western blotting was carried out for the indicated proteins. The knockdown was quantitated by averaging out the TopBP1 levels in the three different siRNA treatment sets (bottom panels). (C) Knockdown of CK2 components compromises the ability of E2-WT to retain ptk6E2-luc. Asterisk (*) indicates a significant difference between this sample and the others on days 6 and 9, *P* < 0.05. Standard error bars are too small to show up on the log scale; results represent the summary of three independent experiments.

**FIG 6:**
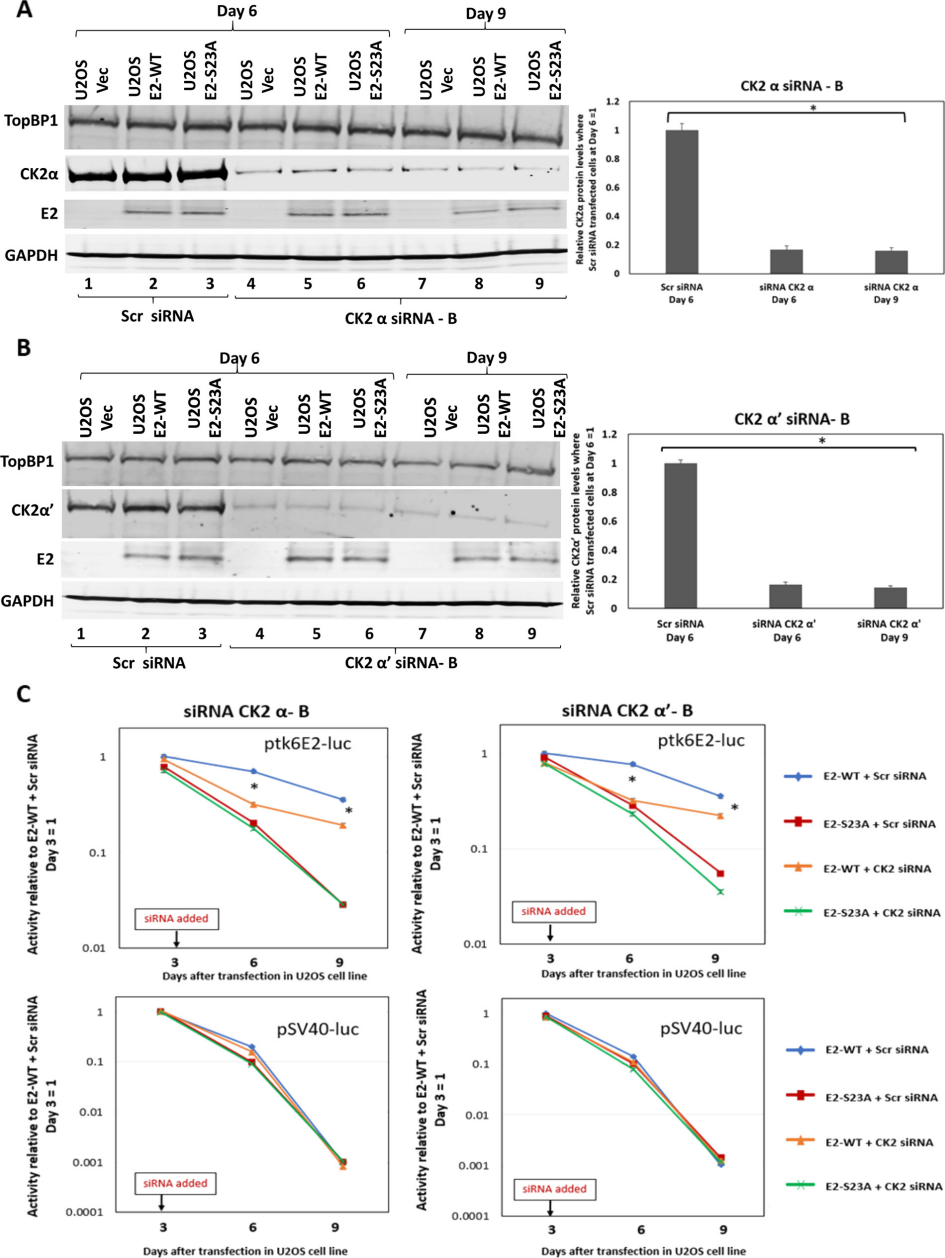
Knockdown of CK2 components disrupts E2 segregation/retention function in U2OS cells. The experiments described in Fig. 6 are a repetition of those shown in Fig. 5, with alternative CK2 component-targeting siRNAs. These results demonstrate that any effects of the siRNAs are not due to off-target effects.

To further confirm that the E2-TopBP1 interaction is require for the E2-WT retention/segregation function, we knocked down components of CK2, a kinase which we demonstrated phosphorylates serine 23 of E2-WT to promote complex formation with TopBP1 *in vitro* and *in vivo* (44). CK2 functions as a tetramer that has two active catalytic components: α and α’ (47). It is not possible to knockout both α subunits simultaneously because cells become unviable; therefore, we knocked out the components individually and measured their effects on E2-WT plasmid retention/segregation function. Knockdown of either component compromises the E2-TopBP1 interaction because it reduces E2 phosphorylation on serine 23, and neither siRNA disrupts the expression of the other α component (44). Figures 5A and B demonstrate statistically significant knockdown of CK2α and CK2α’, respectively, during the time of the assay, without significant disruption to E2 expression. Figure 5C demonstrates that knockdown of CK2α or α’ results in compromised segregation/retention E2 function. Noticeably, on day 6, there was a total elimination of E2 retention function that somewhat recovered by day 9. This is perhaps due to the reorganization of the CK2 enzyme in the cells, arranging into tetramers with only CK2α or α’ components compensating for the loss of the other α component. To confirm that the effects with the CK2α-targeting siRNAs were not due to off-target effects, we repeated these experiments with additional siRNAs and obtained identical results (Fig. 6).

### The E2-TopBP1 interaction is critical for E2 segregation/retention function in human keratinocytes.

**FIG 7:**
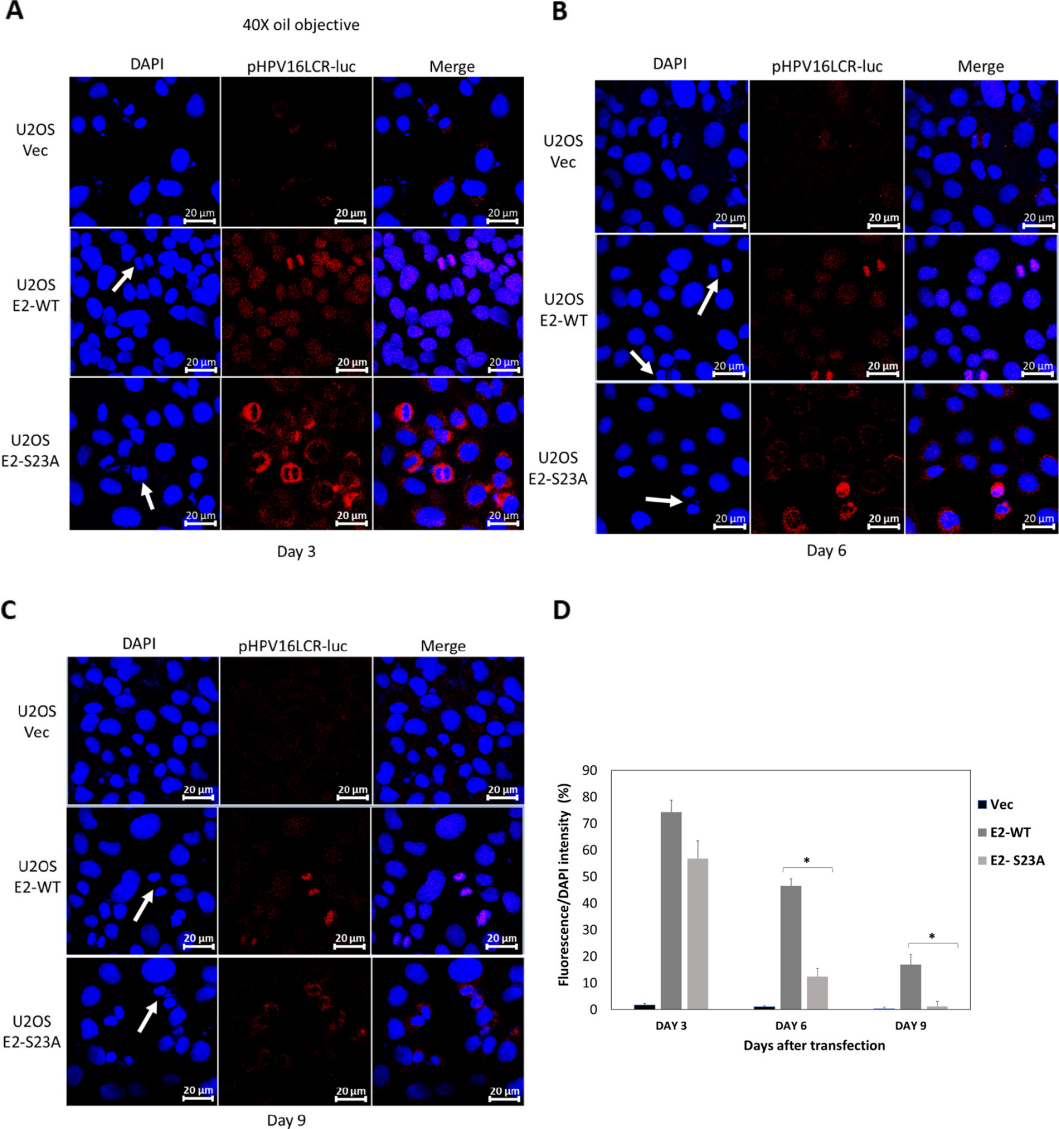
E2 recruits human papillomavirus 16 long control region (HPV16-LCR)-containing plasmids to U2OS cell mitotic chromatin in a TopBP1-interaction dependent manner. (A) Fluorescently labeled pHPV16-LCR-luc was transfected into the indicated cell lines. Three days later, images were captured using a Zeiss LSM700 confocal microscope. (B) Cells used in panel A were trypsinized and re-plated, and images captured 6 days following transfection, as in panel A. (C) The cells used in panel B were trypsinized and re-plated, and images captured 9 days following transfection, as in panel A. Mitotic cells are indicated with white arrowheads in the confocal images. (D) A Keyence imaging system was used to quantitate the fluorescence generated from the transfected plasmid versus that generated from DAPI (4′6′-diamidino-2-phenylindole) staining. Results were generated from three randomly selected fields from each sample. Asterisk (*) indicates that there was a statistically significant difference between E2-WT and E2-S23A samples, *P* < 0.05.

To confirm that the HPV16-LCR (long control region containing transcriptional control elements, the origin or viral replication, and E2 DNA-binding sites) was also segregated by E2 in a TopBP1 interaction-dependent manner, we carried out the following experiment. We could not use our luciferase-based assay because the HPV16-LCR is repressed by both E2-WT and E2-S23A (42). As an alternative, we fluorescently labeled pHPV16-LCR-luc and transfected it into U2OS-Vec, U2OS-E2-WT, and U2OS-E2-S23A, and carried out an experiment similar to that described in Fig. 1 with ptk6E2-luc. Figure 7A demonstrates that 3 days following transfection, there was very little fluorescence detected in U2OS-Vec cells, while there was significant fluorescence detected in both U2OS-E2-WT and U2OS-E2-S23A cells. In U2OS-E2-WT mitotic cells, the fluorescent plasmid is associated with the mitotic DNA, while in U2OS-E2-S23A cells this is not the case (white arrows in the middle panels indicate example mitotic cells). At day 6 following transfection, there remained significant fluorescence in E2-WT and E2-S23A cells, and the fluorescent plasmid only associates with mitotic chromatin in the presence of E2-WT (Fig. 7B). At day 9, there remained easily detectable fluorescence in E2-WT cells (and interaction with mitotic chromatin) while in E2-S23A cells there was some residual fluorescence (Fig. 7C). We quantitated the plasmid fluorescence over the duration of this experiment in 3 randomly chosen fields and expressed the results relative to DAPI (4′6′-diamidino-2-phenylindole) fluorescence (which highlights the cellular DNA), and the results are presented graphically in Fig. 7D. At day 3 following transfection, there was no statistical difference in plasmid fluorescence between E2-WT and E2-S23A cells. However, on both days 6 and 9, there was significantly more fluorescence in E2-WT cells than in E2-S23A cells. Overall, these results demonstrate that E2-WT can segregate and retain plasmids containing the HPV16 LCR, and that the E2-S23A mutant is deficient in this function.

**FIG 8:**
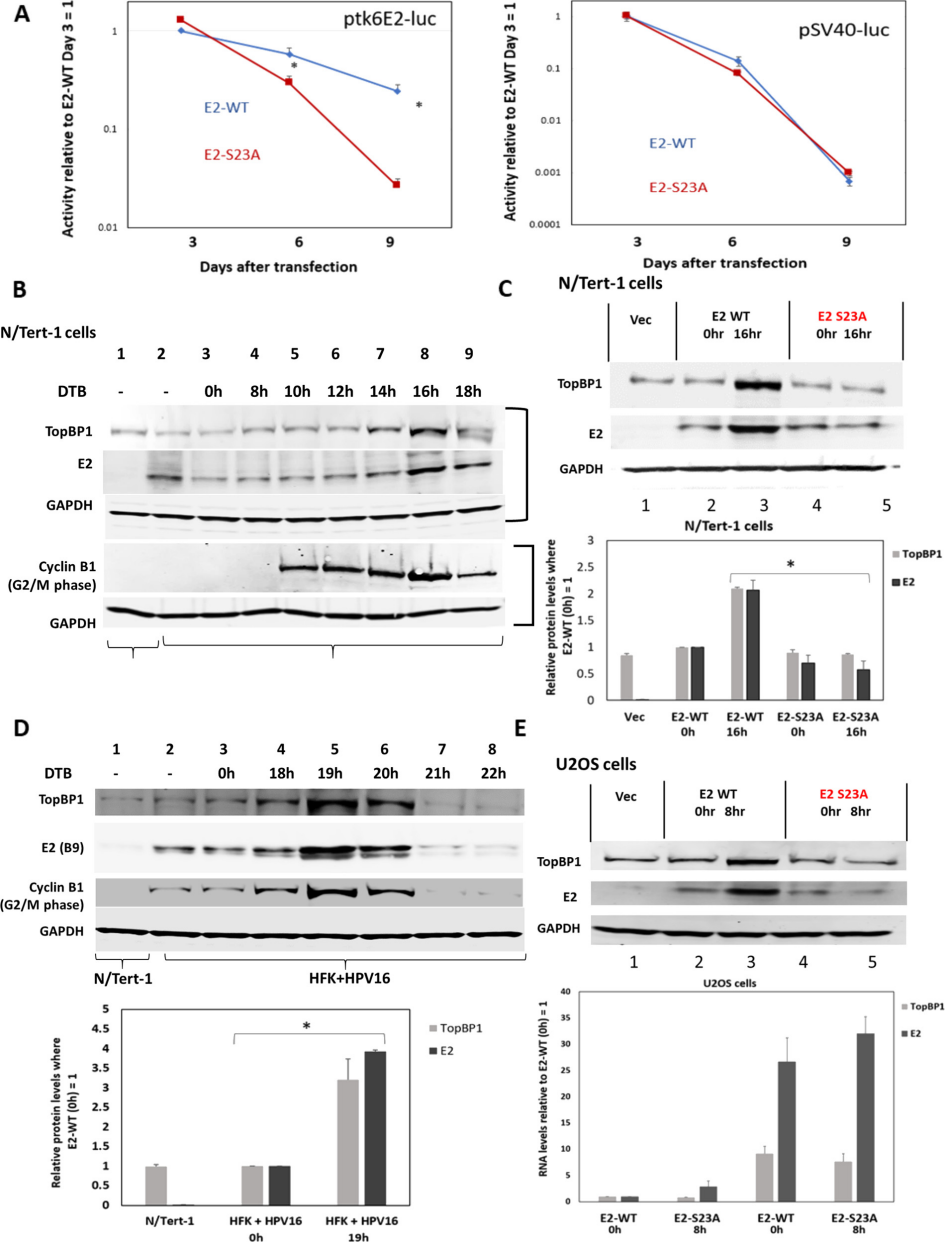
TopBP1 interaction regulates E2 plasmid segregation/retention function and mitotic expression in human keratinocyte cells. (A) Luciferase activity was detected in the indicated N/Tert-1 cells. Day 3 luciferase activity for both E2-WT and E2-S23A was statistically identical (44). For ptk6E2-luc, on day 6, E2-WT retained around 90% of day 3 activity while E2-S23A retained around 15%. On day 9, E2-WT retained around 20% of day 3 activity while most activity was lost with E2-S23A. For pSV40-luc, activity was lost rapidly in both E2-WT and E2-S23A cells. Asterisk (*) indicates a significant difference between this sample and the others at days 6 and 9, *P* < 0.05. Standard error bars are too small to show up on the log scale; results represent the summary of three independent experiments. (B) N/Tert-1 were double thymidine blocked and released at the indicated time points. Harvested proteins were then Western blotted for the indicated proteins, and the results demonstrate a peak of E2 and TopBP1 expression at the 16-h release time point. This corresponds with the peak in expression of the mitotic marker Cyclin B1, indicating that, 16 h following double thymidine block release, there is a significant enrichment of mitotic cells. Results are from two different Western blots from the same extracts, and two GAPDH (glyceraldehyde 3-phosphate dehydrogenase) controls are therefore included. Separate blots are indicated by the brackets to the right-hand side of the figure. (C) N/Tert-1 cells were double thymidine blocked and released for 16 h when the cells are in mitosis (B). Harvested proteins were then Western blotted for the indicated proteins, and the results demonstrate increased expression of E2-WT and TopBP1 during mitosis, but neither E2-S23A nor TopBP1 in the E2-S23A cells. This was repeated and quantitated, demonstrating statistically significant increases in both E2 and TopBP1 in the E2-WT cells compared with that in E2-S23A. (D) Human foreskin keratinocytes immortalized by HPV16 (HFK+HPV16) (44) were double thymidine blocked and released at the indicated time points. Protein extracts were prepared and the indicated Western blottings carried out. Cyclin B indicates G_2_/M phase. Results demonstrate significant increases in both E2 and TopBP1 corresponding with the peak in Cyclin B1 expression (19 h), demonstrating that E2 and TopBP1 levels increase during mitosis in HPV16-positive cells. The experiment was repeated and the duplicates at 19 h post-double thymidine block release demonstrate significant increases in E2 and TopBP1 during mitosis. (E) As reported previously, E2-WT, but not E2-S23A, protein levels are increased in mitotic U2OS cells (44). We harvested RNA from repeat experiments at the indicated time points (lower panel) and demonstrated that there was no significant difference between E2-WT and E2-S23A RNA levels during mitosis. Therefore, any changes in E2 protein levels are due to post-transcriptional events.

Next, we wanted to determine whether the plasmid segregation function of E2-WT functions in human keratinocytes. Using N/Tert-1 cells stably expressing E2-WT or E2-S23A (44), we carried out our luciferase segregation/retention assay. Figure 8A demonstrates that in N/Tert-1 cells, E2-WT is able to retain ptk6E2-luc activity compared with that in E2-S23A cells, similarly to U2OS cells (Fig. 3). E2-WT protein expression levels in U2OS increase during mitosis, and TopBP1 levels also increase during mitosis in the presence of E2-WT (42). Neither E2 nor TopBP1 levels increase in E2-S23A cells. To identify a mitotic cell population in N/Tert-1 cells, they were released from a double thymidine block and harvested at 2-h intervals from 8 h post-release. We chose this time point because N/Tert-1 cells visibly grow slower than U2OS cells which are in mitosis 8 h following release (44), therefore we assumed that N/Tert-1 cells would not enter mitosis prior to 8 h. We blotted for the mitotic marker Cyclin B1 and demonstrated that the peak of mitosis is around the 16-h time point (where there is maximum Cyclin B1 expression, Fig. 8B). We therefore used 16 h following double thymidine blocking release as a N/Tert-1 cell population enriched for mitotic cells and repeated and quantitated E2 and TopBP1 expression at this time point; there was a significant increase in both E2-WT and TopBP1 protein expression in mitotic N/Tert-1 cells. There was no increase in E2-S23A or TopBP1 in the E2-S23A expressing cells (Fig. 8C). Next, we wanted to determine whether this increase in E2 protein expression during mitosis occurs in cells immortalized by the HPV16 genome. Figure 8D demonstrates that this is the case. Human foreskin keratinocytes immortalized with HPV16 genomes (HFK+HPV16) were double thymidine blocked (DTB) and released at the indicated time points; this cell line has been described previously (44). Preliminary studies to help identify mitosis indicated that there was no change in E2 or TopBP1 expression levels before a 16-h time point following release from the block (data not shown). In Fig. 8D, Cyclin B1 antibody confirms that the increases in E2 and TopBP1 levels occur during mitosis. N/Tert-1 cells, an hTERT-immortalized human foreskin keratinocyte cell line (lane 1), were used as a control demonstrating the specificity of the E2 antibody. Finally, we addressed the issue of whether the increase in E2 expression is due to an increase in RNA (Fig. 8E). This figure demonstrates the E2-WT specific increases in E2 and TopBP1 compared with E2-S23A in U2OS cells (top panel), as reported previously (44). The bottom panel demonstrates that both E2 and TopBP1 RNA levels increase during mitosis irrespective of E2-WT or E2-S23A expression, but that there was no significant difference between the E2 and TopBP1 RNA levels in the two different E2-expressing cell lines. This demonstrates that the increase in E2-WT and TopBP1 protein expression during mitosis, compared with that in E2-S23A cells, is a post-transcriptional event.

## DISCUSSION

While E2 interaction with mitotic chromatin suggests that it is a plasmid segregation/retention factor during the HPV16 life cycle (43), to date, there is little evidence that E2 performs such a function. To our knowledge, only one study has described assays for measuring E2 segregation/retention function, and that was for BPV1 E2 (48). In this assay, green fluorescent protein (GFP) expression was monitored in a plasmid that contained the GFP gene, E2 DNA-binding sites, and an E2 expression cassette. This study indicated there were two functions of E2 contributing to the plasmid segregation/retention function of BPV1 E2, interaction with mitotic chromatin by itself was insufficient. We are able to generate stable cells expressing E2-WT and mutant proteins enabling us to dissociate E2 expression from reporter plasmids used in segregation/retention assays (18, 44, 49–51). We exploited these cell lines to demonstrate that E2 protein has plasmid segregation/retention function and that this activity is dependent upon the E2 interaction with TopBP1. Figure 1 demonstrates that ptk6E2-luc is recruited to mitotic chromatin by E2-WT, but not by the TopBP1 interaction-deficient E2-S23A. E2-WT retains fluorescent signals 9 days following transfection, while E2-S23A does not. We demonstrate that the retention of fluorescent ptk6E2-luc was not due to integration via removal of the fluorescent signal via a salt wash. Figure 3 shows exploitation of the ptk6E2-luc plasmid to develop a quantitative assay for measuring E2 segregation/retention function, and this again demonstrates that E2-S23A has lost this function compared with E2-WT. To confirm that the failure of E2-S23A in these assays to segregate/retain is due to a failure to interact with TopBP1, we took two approaches. First, as shown in Fig. 4, we used siRNA to knock down TopBP1 expression, resulting in a loss of the segregation/retention function of E2-WT. It is also noticeable that knockdown of TopBP1 resulted in a failure of E2 to interact with mitotic chromatin, correlating with the loss of plasmid segregation function. We repeated this using an additional TopBP1 siRNAs and generated identical results. Second, Fig. 5 and 6 demonstrate that siRNA knock down of CK2 components disrupted E2-WT segregation/retention function. Previously, we demonstrated that CK2 phosphorylation of E2 on serine 23 is required for E2-TopBP1 complex formation (42). To confirm that this plasmid segregation/retention function of E2-WT was not an artifact of ptk6E2-luc, we carried out fluorescent plasmid retention studies with pHPV16-LCR-luc and demonstrated that this plasmid was also segregated/retained by E2-WT but not by E2-S23A in U2OS cells. Altogether, these results demonstrate that E2-WT has segregation/retention function which is mediated via interaction with TopBP1.

All of the results discussed above were carried out in U2OS cells. We moved this project into human keratinocytes, the natural target cell for HPV16 infection and disease. Previously, we demonstrated that in N/Tert-1 cells (human foreskin keratinocytes immortalized with hTERT), E2-WT interacted with TopBP1, while E2-S23A could not (44). We also demonstrated that CK2 phosphorylated E2 on serine 23 in N/Tert-1 cells (44). Figure 8 demonstrates, using our luciferase-based plasmid segregation/retention assay, that E2-S23A has lost the ability to mediate this function, just as it has in U2OS cells. We previously reported that E2 and TopBP1 protein levels increased during mitosis in U2OS cells in an interaction-dependent manner, and we demonstrate that this is also true in N/Tert-1 cells (Fig. 8). This increase in E2 and TopBP1 levels during mitosis also occurs in HFK+HPV16 cells (Fig. 8).

Overall, the novel assays described here confirm that E2 has plasmid segregation/retention activity, that an interaction with TopBP1 is critical for this function, and that the E2-TopBP1 interaction is also critical for this function in human keratinocytes. The stabilization of E2 during mitosis in HFK+HPV16 cells also suggests that E2 can carry out plasmid segregation and retention during the viral life cycle. We are currently investigating the mechanism of E2 protein increase during mitosis, as abrogation of this increase would disrupt the HPV16 life cycle. Previously, we demonstrated that increased levels of E2 are mediated by enhanced acetylation, and this is a current focus of our studies (52). There are likely other factors involved in the E2-TopBP1 mitotic complex which are involved in mediating the segregation/retention function. For example, we recently demonstrated that TopBP1 and BRD4 form a complex and we are currently investigating the role of this interaction in the plasmid segregation function of E2 (44). Additionally, ChlR1 and SMC6 have also been implicated as being involved in E2 interaction with mitotic chromatin and therefore plasmid segregation function (53, 54). Much work remains to fully understand the host complex that controls E2 interaction with mitotic chromatin and mediates plasmid segregation function, but this report demonstrates that the interaction between TopBP1 and E2 is critical for E2 segregation function. TopBP1 is also important during mitosis as it repairs and replicates DNA, and therefore the change in TopBP1 levels and localization (recruitment to mitotic chromatin) mediated by E2 may generate vulnerabilities to mitotic poisons in HPV16-positive cells (35–37, 39–41, 55, 56). We are currently investigating this possibility.

## MATERIALS AND METHODS

### Cell culture.

Stable cell lines expressing wild-type E2 (E2-WT) and E2-S23A (E2 with serine 23 mutated to alanine, abrogating interaction with TopBP1), alongside cells with pcDNA empty vector plasmid control, were established both in U2OS and N/Tert-1 cell lines as previously described (18, 44). Cell cultures were performed as described previously. Immortalization and culture of human foreskin keratinocytes with HPV16 E2-WT was performed as previously described (44).

### Generation of fluorescently tagged plasmids and transfection.

Label IT Tracker (Mirus Bio, cat no. MIR7025) protocol was used to covalently attach a fluorescein-492 tag to ptk6E2-luc, a plasmid containing 6 E2 DNA-binding sites upstream from the luciferase gene controlled by the tk promoter (45). This fluorescent ptk6E2-luc plasmid was then transiently transfected into following stable cells: U2OS-Vec (vector control), U2OS-E2-WT (stably expressing wild type E2), and U2OS-E2-S23A. At 48 h after transfection, the cells were passaged for the next time point and a separate set of cells grown on coverslips were simultaneously fixed, stained with DAPI, and observed for the presence of the fluorescent ptk6E2-luc or pHPV16-LCR-luc (31). Images were captured using a Keyence imaging system (BZ-X810) or a Zeiss LSM700 laser scanning confocal microscope. The intensities of the fluorescently tagged plasmids were measured relative to DAPI at three different regions using the Keyence BZ analyzer software.

### Immunofluorescence.

U2OS cells expressing stable E2-WT, E2-S23A, and pcDNA empty vector plasmid control were plated on acid-washed, poly l-lysine-coated coverslips in a 6-well plate at a density of 2 × 10^5^ cells/well (5 mL Dulbecco’s modified Eagle’s medium [DMEM] + 10% fetal bovine serum [FBS] medium). After 48 h of transfection, the cells were washed twice with phosphate-buffered saline (PBS), fixed, and stained as previously described (44). The primary antibodies used are as follows: HPV16 E2 B9 monoclonal antibody, 1:500 (44); and TopBP1, 1:1,000 (Bethyl Laboratories, cat no. A300-111A). The cells were washed and incubated with secondary antibodies Alexa Fluor 488 goat anti-mouse (Thermo Fisher, cat no. A-11001) and Alexa Fluor 594 goat anti-rabbit (Thermo Fisher, cat no. A-11037) diluted at 1:1,000. The wash step was repeated, and the coverslips were mounted on a glass slide using Vectashield mounting medium containing DAPI. Images were captured using a Keyence imaging system (BZ-X810) or a Zeiss LSM700 laser scanning confocal microscope and analyzed using Zen LE software.

### Western blotting.

Protein was harvested from cell pellets lyzed using 2× pellet volume NP-40 lysis buffer (0.5% Nonidet P-40, 50 mM Tris [pH 7.8], and 150 mM NaCl) supplemented with protease inhibitor (Roche Molecular Biochemicals) and phosphatase inhibitor cocktail (Sigma-Aldrich). The cells were lyzed for 20 min on ice followed by centrifugation at 18,000 rcf (relative centrifugal force) for 20 min at 4°C. Protein concentration was estimated colorimetrically using a Bio-Rad protein assay. Next, 50 μg of protein with equal volume of 4× Laemmli sample buffer (Bio-Rad) was denatured at 95°C for 5 min. Proteins were separated using a Novex WedgeWell 4% to 12% Tris-glycine gel (Invitrogen) and transferred onto a nitrocellulose membrane (Bio-Rad) using the wet-blot method at 30 V overnight. The membrane was blocked with LI-COR Odyssey blocking buffer (PBS) diluted 1:1 vol/vol with PBS and then incubated with specified primary antibody in LI-COR Odyssey blocking buffer (PBS) diluted 1:1 with PBS. Next, the membrane was washed with PBS supplemented with 0.1% Tween 20 and probed with the Odyssey secondary antibodies (IRDye 680RD Goat anti-Rabbit IgG [H+L] 0.1 mg or IRDye 800CW Goat anti-Mouse IgG [H+L] 0.1 mg) in LI-COR Odyssey blocking buffer (PBS) diluted 1:1 with PBS at 1:10,000 for 1 h at room temperature. After washing with PBS-Tween, the membrane was imaged using the Odyssey CLx Imaging System and ImageJ was used for quantification of protein band intensities standardized to GAPDH (glyceraldehyde 3-phosphate dehydrogenase) levels. Primary antibodies used for Western blotting studies are as follows: monoclonal B9 1:500 (44), TopBP1 1:1,000 (Bethyl, cat no. A300-111A), GAPDH 1:10,000 (Santa Cruz; cat no. sc-47724), casein kinase IIα (1AD9) 1:500 (Santa Cruz, cat no. sc-12738), CKII alpha' antibody 1:1,000 (Bethyl, cat no. A300-199A), and Cyclin B1 (D5C10) XP Rabbit MAb 1:1,000 (Cell Signaling Technology, cat no. 4138).

### Plasmid segregation assay.

Two luciferase reporter plasmids were used for our novel assay: one containing the SV40 promoter and enhancer (pGL3 Control, Promega, described as pSV40-luc in the manuscript), which has no E2 DNA-binding sites; the other with the HSV1 tk promoter driving expression of luciferase with 6 E2 target sites upstream (see results for details). The pSV40-luc and ptk6E2-luc were transiently transfected, separately, into either E2-WT or E2-S23A cells. Three days post-transfection, the cells were trypsinized, with half re-plated and half harvested for a luciferase assay system (Promega). This luciferase activity was the baseline activity. At day 6, the same process was repeated: half of the cells harvested for luciferase assay, half re-plated. At day 9, cells were harvested for luciferase activity assay. A Bio-Rad protein estimation assay was used for protein concentration estimation to standardize for cell number. Relative fluorescence units were measured using the BioTek Synergy H1 hybrid reader. The activities shown are expressed relative to the respective protein concentrations of the samples. The assays shown are representative of three independent experiments carried out in triplicate.

### Small interfering RNA and segregation assay.

U2OS parental cells were plated on 100-mm plates. The next day, cells were transfected using 10 μM siRNA listed at the end of this paragraph. Ten μM MISSION siRNA Universal Negative Control (Sigma-Aldrich, cat no. SIC001) was used as a “non-targeting” control in our experiments. Lipofectamine RNAiMAX transfection (Invitrogen; cat no. 13778-100) protocol was used in the siRNA knockdown. At 48 h post-transfection, the cells were harvested and knockdown confirmed by immunoblotting for the protein of interest. Segregation assays were performed as described previously, after treating the cells with the siRNA of interest on day 3 of the protocol. All siRNAs were purchased from Sigma-Aldrich: siRNA TopBP1-A CUCACCUUAUUGCAGGAGAdTdT; TopBP1-B GUAAAUAUCUGAAGCUGUAdTdT, CK2α-A GGCUCGAAUGGGUUCAUCUtt; CK2α-B GAUGACUACCAGCUGGUUCdTdT, CK2α’-A CAGUCUGAGGAGCCGCGAGdTdT, and CK2α’-B AUACAGCCCAAACUCCACAUUU.

### Cell synchronization.

U2OS and N/Tert-1 cells expressing stable E2-WT, E2-S23A, and pcDNA empty vector plasmid control were plated at 3 × 10^5^ density onto 100-mm plates in DMEM + 10% FBS medium. The cells were treated with 2 mM thymidine diluted in the supplemented DMEM for 16 h. Cells were then washed 2 times with PBS and recovered in supplemented DMEM. After 8 h, to block the cells at G_1_/S phase, a second dose of 2 mM thymidine was added and the cells were incubated for 17 h. The cells were then washed twice with PBS and recovered as before at the following time points. For U2OS, cells were harvested at 0 h (G_1_/S phase) and 8 h (M1 phase). For N/Tert-1, cells were harvested at 0 h (G_1_/S phase) and 16 h (M1 phase). The cell lysates were prepared using the harvested cells at the time points mentioned, and immunoblotting was carried out. The above procedure was repeated in HFK cells immortalized with HPV16 genomes (HFK+HPV16 E2-WT) grown with 3T3-J2 fibroblasts. N/Tert-1 vector control cells were used as controls. Cyclin B1 antibody was used to confirm the mitosis phase in these cells using immunoblotting.

### SYBR Green quantitative reverse transcription PCR.

Following cell synchronization, RNA was isolated from the harvested cells using the SV Total RNA isolation system (Promega) according to manufacturer’s instructions. Two μg of RNA was reverse-transcribed into cDNA using a high-capacity reverse transcription kit (Applied Biosystems). cDNA and gene-specific primers were added with PowerUp SYBR Green Master Mix (Applied Biosystems), and real-time PCR was performed using a 7500 Fast Real-Time PCR system. Expression was quantified as relative quantity over GAPDH using the threshold cycle (2^−ΔΔCT^) method. The primer sequences utilized were as follows: HPV16 E2 forward, 5′-ATGGAGACTCTTTGCCAACG-3′; HPV16 E2 reverse, 5′-TCATATAGACATAAATCCAG-3′; TopBP1 forward, 5′-TGAGTGTGCCAAGAGATGGAA-3′; TopBP1 reverse, 5′-TGCTTCTGGTCTAGGTTCTGT-3′; GADPH forward, 5′-GGAGCGAGATCCCTCCAAAAT-3′; GAPDH reverse, 5′-GGCTGTTGTCATACTTCTCATGG-3′.

### Statistical analysis.

Segregation assay and quantitation of Western blots and fluorescence/DAPI intensity results are represented as mean ± standard error (SE). A Student’s *t* test was used to determine significance.
